# Loot

**Published:** 1873-05

**Authors:** 


### Loot.

### *Elevating the Standard.*

Our friend of the Medical Record thinks it must be a very poor medical school which would not, in twenty years, turn out one or more who would afterwards work out for themselves a reputation that would not die with them. We dare say he is right in this; for it would be difficult to believe that a road-side public house could exist prosperously for a score of years without enabling the landlord to show on his register names whose owners had risen to celebrity after supping and lodging with him. But we think it extremely doubtful whether a landlord, in all the country round, would swallow metaphysics enough in a lifetime to attempt to establish the relation of cause and effect between his jug-tavern and that celebrity. Oh! no! Nobody but a doctor could do that.

Let us be understood. There were great doctors before medical schools, and there have been great ones since, and in despite of them. We believe a medical school never made one great man, and never will, though they are helps and spring-boards in that direction.

The new plan to force this unnatural relation, by adding to the name of the graduate the place where he was born into medicine, in the vain hope that mamma may help baby along, to say nothing of the palpable violation of our ethics it involves, is a laughable puerility. One signs himself P. Q. Z., M.D., Edin., and immediately there spring up, in the mind of the reader, winding rivers, tall, thin-clad trees, long grass, and abundance of sunshine and azure, with a couple of fig-leaf-aproned people, otherwise nude, of opposite sexes, and desperately in love with each other. But unfortunately, Eden is spelled with an "i," and the picture vanishes. What can it mean? Why, it is intended to convey the idea that the owner of that name graduated at Edinburgh, Scotland—a weak parody upon "Help me, Cassius, ere I sink." Another writes "Harv." at the end of his name, to show that mamma is somebody, whatever may be thought of young hopeful. But the reader seeing "Harv.," has his mind running on "harvest home," and is busy recalling its festivities with the English peasants in the time of Henry VIII. Then comes "Jeff.," and "Penn.," and "Belle," and "Lou.," and "Cin.," and "Nash.," and a whole Babel of jargon of thundering sound, signifying nothing—non-asinine.

Graduate in Medicine from our honored old University on yonder hill, rely on nothing under God but your own brave soul, with fixed determination of purpose to *deserve* distinction, and, if life lasts, it will perch upon the standard that *you*, not I, have raised. Here, as elsewhere, you must work out your own salvation.

> I plant my standard in the milky way,
> Upon the very key-star of the arch,
> And to my hesitating pupil say,
> "Look ye steadfast, upward, and forward, march!"
>
> Let its bright silken streamers rustle there,
> Amid the gems and glories of the sky.
> While pointing to it, in that virgin air,
> I ask opposers, "How is that for high?"

—*Nashville Jour. Med. and Surg.*

### *Attempted Reduction of Dislocation of Shoulder Joint—Rupture of Axillary Artery—Death.*

The patient was admitted into the Royal Infirmary, Edinburgh, under the care of Mr. Lister, on account of an unreduced dislocation of the shoulder joint of seven weeks' standing, but said by the patient himself to have been of only five weeks' duration. Professor Lister, by manipulation, and subsequently by the pulleys, attempted to reduce the dislocation, no undue force being exerted by either method. During the attempt a sharp crack was heard, and subsequently a swelling, which ultimately reached the size of an adult head, took place on the dorsal and posterior part of the scapula; the occurrence being due to the rupture of the axillary artery, and subsequent effusion of blood into the surrounding textures.

Without hesitation, Professor Lister cut down on the spot, and proceeded to search for the ruptured vessel, opening, by his incision, into a sacculated structure into which the blood had been effused, and from the bottom of which the blood appeared to flow. Careful search for the source of the hemorrhage was, after a considerable period of time, rewarded, by finding that the bleeding-point was situated on the posterior aspect of the artery itself—a situation which, the Professor remarked, was rather unusual, and not likely to be easily got at or seen. The vessel was accordingly ligatured above and below the bleeding point, antiseptic measures being taken during and after the operation to prevent the occurrence of putrefaction. The patient rallied immediately after the operation, but sank and died in about three hours subsequently.

Professor Lister remarked on the importance of the occurrence from a practical point of view, at the same time remarking on the obvious duty of at once cutting down and securing the bleeding vessel.

A dissection of the parts showed several features of interest. The humerus had attached to its staff a little spiculum of bone, which was evidently the immediate cause of the perforation of the artery, which itself was highly atheromatous, and so rendered the more friable. The head of the bone had, during the comparatively short period of seven weeks, formed for itself a false glenoid cavity, in which it had been reposing; the existence of this structure forming an additional element in the difficulty of successfully or readily reducing the dislocation. Short as the period had been, it was instructive (said Professor Lister) to note that this cavity was not only of cartilaginous texture, but even exhibited traces of osseous development also.

Professor Lister, in conclusion, regretted that he had not instructed his house-surgeon to send for him at once on the intervention of the serious later symptoms, as the case would have presented the most favorable aspects for the performance of the operation of transfusion of blood from the veins of a person willing to furnish the requisite supply.—*Med. Times and Gazette*, Feb. 1, 1873.—*Medical News.*

### *What is Homœopathy?*

According to the census of 1870, unless our information is wholly incorrect, the proportion of homœopathic practitioners was a fraction over seven per cent. of the whole body of the medical profession in the United States. Be it remembered that this is after over thirty years of effort to propagate the doctrines of that peculiar school, and in spite of a strong tendency on the part of physicians everywhere to investigate, and to adopt, if satisfactory, whatever new principle or practice promises to aid them in the battle with disease. Can it be, then, anything but the warmest enthusiasm that enables an orator before the New York State Homœopathic Medical Society to say, "Who will deny that, at no distant date, either under the name of the Homœopathic School, or adopting a title now often used synonymously,—that of the 'Modern School of Rational and Liberal Medicine,'—we may see the two great branches of the medical profession, for many years estranged and often hostile, united in one solid phalanx, under the ample folds of that banner, unfurled to the gaze of an astonished world by the genius of Samuel Hahnemann, on which, inscribed in letters of living light, are the words '*similia similibus curantur*,' and, actuated by that philosophic and Christian motto, *in certis unitas, in dubiis libertas, in omnibus charitas*,' marching onwards to the attainment of conquests over disease and death, such as have never yet been depicted in the dreams of the most ardent medical enthusiast? Then will have come the millennium of medicine. God grant a consummation so devoutly to be wished!"

No doubt the homœopathists would be glad to have the profession at large adopt their absurd dogma, to which they themselves, in their practice, do not adhere. No doubt they would be glad to have us, with all the results of scientific research, and the records of past battles gallantly won or as gallantly lost, come over to their ranks. But why do they not show us one single case in which a remedy produces, in the healthy body, the genuine symptoms of a disease in which it is known to be useful? Why do they not show us that belladonna will induce something more than the most superficial and unimportant phenomena of scarlet fever? that chloral or the bromide of potassium will prevent sleep? that mercury or iodide of potassium will give rise to the essential secondary or tertiary manifestations of syphilis? How do they explain the perversity with which the entire scientific world declines to recognize them? We cannot suppose that Humboldt, Arago, Sir Humphrey Davy, Agassiz, and a host of others—dead and living—men of inquiring minds, intolerant of any bar to the freest examination of every theory or hypothesis in regard to the secrets of nature,—would have hesitated for one moment to give in their allegiance to any doctrine, if it could maintain itself by facts.

From our own free wanderings in the field of homœopathic literature, we are convinced that the more extensively regular physicians are acquainted with it, the more strengthened will they be in their sense of its absurdity. Even the advocates of that dogma find that their own school affords them insufficient nutriment; for in a recent number of one of their leading journals, out of thirty quoted items, *twenty-five* were from regular medical sources, *three* only from homœopathic; one was from a secular paper, and one was not credited at all.

We answer the question which heads this article, by asserting that homœopathy is a mere name, signifying nothing.—*Philad. Med. Times.*

***The Horse-Influenza.***

Our readers will have had such a surfeit of items in the daily papers with regard to the influenza now prevailing among animals of the horse kind, that we propose only to call attention to a few of its more remarkable features.

In the first place, the singular rapidity of its progress precludes the idea of its spread by contagion. It was first known in Canada, less than six weeks ago, and since that time has spread over the whole of New York, and the Eastern States, and Pennsylvania, and is now (November 9) felt in Baltimore. Only if very large numbers of horses were moving in this southward direction would the theory of its propagation in this way be at all tenable. Then, again, it has affected all the horses, in small villages and in the country. Some atmospheric convection, independent of wind-currents, must necessarily be the means of its spread.

Secondly, the universality of the disease is to be noted: we may perhaps say that not a single horse has escaped, as even those which have not been actually laid up have shown the effects of its depressing influence. Mules seem less susceptible to it than horses, but many of them, and we believe all more or less, have felt it. Within the memory of the oldest horseman there has never been such a visitation; and indeed the peculiar inconveniences entailed are such as would have impressed the recollection of an epizootic of the kind upon the minds of the public as well.

Thirdly, the exemption of all but animals of the horse kind. There have, it is true, been a few cases of influenza in the human species, but the health of the city may be said to be exceptionally good, and the mortality returns are very small. The vague rumors of horned cattle being affected, which have prevailed from time to time, are not confirmed so far.

The deaths seem to have been limited to such horses as were previously in poor condition, from age, over-work, or disease; some also, from neglect in the early stages of the epizootic, have been attacked with fatal pneumonia. It may be that, like the poison of scarlatina or small-pox, the peculiar cause of this disorder is in some cases so virulent as to hurry off the animals affected before the symptoms are fully developed; but of this the evidence is not yet satisfactory. Another cause of death has doubtless been the working of horses before they had fully recovered their strength, congestion of the lungs being especially apt to occur under such circumstances, and to run on into fatal pneumonia.

We trust that from the intelligent and careful investigation of this remarkable scourge, there may be derived some facts bearing upon the natural history and mode of propagation of the zymotic diseases which affect the human family.—*Ibid.*

